# THE MODERATING ROLE OF PURPOSE IN LIFE IN THE RELATIONSHIP BETWEEN DISCRIMINATION AND COGNITION

**DOI:** 10.1093/geroni/igad104.0077

**Published:** 2023-12-21

**Authors:** Heather Farmer, Alexis Ambroise

**Affiliations:** University of Delaware, Newark, Delaware, United States; University of Delaware, Newark, Delaware, United States

## Abstract

Discrimination is a source of chronic stress disproportionately experienced by Black adults that has been associated with worse cognitive outcomes in older adults. Few studies have explored modifiable resilience resources that may protect people from the possible negative impacts of discrimination on cognition. Sense of purpose refers to an individual’s perception of future-oriented goals and direction in life, and is a key resource associated with better cognition. However, no studies have explored whether greater purpose may buffer older adults against the negative impacts of discrimination on cognition or whether there are race differences in the stress-buffering impact of purpose on cognition. We use data from 9,775 Non-Hispanic White and 1,687 Black adults in the 2006 to 2016 waves of the Health and Retirement Study (HRS) to explore whether purpose in life moderates the association between everyday discrimination and cognitive functioning and whether this relationship varies by race. Discrimination was measured using the Everyday Discrimination Scale, which assesses how frequently an individual reports unfair and differential treatment (range, 0-5, where higher scores indicate more frequent discrimination). Multilevel linear regression models revealed that frequent discrimination was associated with worse cognition (b=-0.34, p<.001), controlling for age, gender, race/ethnicity, region, and urbanicity. There was a significant discrimination x purpose interaction (b=0.16, p<.001), suggesting that purpose may buffer the discrimination-cognition association. However, there was a non-significant discrimination x purpose x race interaction. This research suggests a strong sense of purpose can protect people from experiencing worse cognition when faced with frequent discrimination.

